# Morphometric analysis of sinus depth in the posterior maxilla and proposal of a novel classification

**DOI:** 10.1038/srep45397

**Published:** 2017-03-24

**Authors:** Florian Wagner, Gabriella Dvorak, Stefan Nemec, Peter Pietschmann, Hannes Traxler, Kurt Schicho, Rudolf Seemann

**Affiliations:** 1 University Clinic for Cranio- and Maxillofacial Surgery, Medical University of Vienna, Austria; 2 Department of Conservative Dentistry and Periodontology, School of Dentistry, Medical University of Vienna, Sensengasse 2a, 1090 Vienna, Austria; 3 University Clinic for Radiology, Medical University of Vienna, Waehringer Guertel 18-20, 1090 Vienna, Austria; 4 Centre for Pathophysiology, Infectiology and Immunology, Medical University of Vienna, Austria; 5 Department of Systematic Anatomy, Medical University of Vienna, Währinger Strasse 13, 1090 Vienna, Austria

## Abstract

The aim of this study was to analyse the posterior maxillary sinus based on its extension into the alveolar process and to provide a simple clinical classification. A retrospective cohort study was conducted in CT scans of 200 dentate and 200 edentulous patients (100 women and 100 men, respectively). After manual placement of 12 reference points morphometric analysis was performed and sinus depth, residual alveolar ridge height (RH) and the sinus opening angle were calculated. Sinuses were classified according to the quartiles of sinus depth: class I (above the hard palate), class II (0–6 mm below the hard palate) and class III (>6 mm below the hard palate). Sinus depth was found to be a reliable anatomical landmark and did not vary significantly between gender (p = 0.8940) or dentition groups (p = 0.9723). Alveolar height varied significantly between sinus classes (p < 2 × 10^−16^) and dentition groups (p < 2 × 10^−16^) but not between genders (p = 0.5178). The sinus opening angle was significantly different between sinus classes (p < 2.2 × 10^−16^) but not between gender or dentition groups. We propose a novel classification built upon the quartiles of sinus depth, dividing the sinuses into three classes. Our classification is the first one that represents the anatomy of the patient independent of gender and dentition.

Residual ridge resorption (RRR) after tooth loss is the combined result of complex remodelling processes, resulting in changes of the maxillary sinus and shape of the alveolar ridge. These morphometric changes have a big impact on the subsequent preprosthetic treatment and thus have been in the focus of research since the early days of implant therapy in dentistry. Several classifications based on radiological features or on bone morphometry have been proposed since in order to simplify the description of these conditions and to facilitate the communication between clinicians^1^,^2^,^3^,^4^,^5^,^6^,^7^. Cawood and Howell evaluated the morphometric changes occurring in the edentulous jaws in 300 dried skulls and created their well-known classification; they divided the maxillae according to the observed changes in shape of the alveolar process into six classes, ranging from Class I (dentate) to Class VI (depressed ridge form)^1^. Chan *et al*. and Teng *et al*. used the average sinus width between the lateral and the medial wall at sites relevant to sinus augmentation to build their classifications, providing information for the difficulty and the adequate approach for sinus augmentation as well as indications for a preoperative CBCT scan^2^,^5^. Both studies similarly classified the sinuses into narrow, average and wide and found narrow and wide sinuses to be more challenging in sinus augmentation procedures, emphasizing the value of a preoperative CBCT scan. Wang and Katranji finally reviewed the classification systems by Misch *et al*. and Simion *et al*. and created a novel ABC - classification, implicating treatment guidelines under the assumption that implants with at least 4 mm in diameter and 10 mm in height will be placed^7^,^8^,^9^. Sinuses were divided into three classes (“A” – abundant bone, “B” – barely sufficient bone and “C” – compromised bone); while class A sinuses allow for immediate implant placement, class B sinuses require prior vertical augmentation via either osteotome or lateral window approaches. Class C sinuses require extensive vertical and horizontal augmentation via the lateral approach.

The previously mentioned sinus classifications neglect the extension of the maxillary sinus into the alveolar process, which is of crucial knowledge in sinus augmentation procedures. Hence, the aim of this study was to provide a simple clinical classification of the maxillary sinus based on its extension into the alveolar process.

## Materials and Methods

A retrospective cohort study in 400 CT scans was performed according to the Declaration of Helsinki and the Good Clinical Practice (GCP) guidelines after approval of the institutions ethics committee was obtained (No. 1464/2013). The results of this study are reported according to the STROBE criteria^10^.

### Subjects

CT scans used in this study were obtained from randomly selected patients who received a CT scan during clinical routine between October 2007 and November 2013 for various reasons. The most frequent indications for CT scans were tumour follow-up or staging examinations, trauma or suspicion of abscesses. None of the CT scans were solely performed for the purpose of this study. In order to be included into this study patients had to be at least 18 years of age and both sinuses had to be displayed completely. CT scans showing signs of sinus pathologies, signs of operations in the sinus region as well as artefacts impeding assessment were excluded from this study. CT scans showing sinuses with severe mucosal swelling as well as pathologies apart from the maxillary sinus were not excluded from this study. All of the CT scans were extracted from the IMPAX database (Agfa HealthCare GmbH, Bonn, Germany) at the Department of Radiology, Medical University Vienna according to the inclusion criteria until the number of 400 CT scans (200 men and 200 women), respectively, was reached.

### Computed Tomography

All of the CT scans were conducted using a 64-multidetector row CT scanner (Philips Brilliance CT 64, Philips Healthcare, Netherlands). All of the patients were positioned in the supine position with the arms placed parallel to the chest. The imaging parameters were adjusted at 120 kV tube voltage and 175 mAs current-time product. The tube rotation time was set at 0.75 s and the pitch was 0.89. The acquisition collimation of the CT scan was 64 × 0.625 mm and the field of view ranged from 250 to 300 mm in diameter. The CT scans were reconstructed with a slice thickness of 1.5 mm using a bone algorithm (W3000; L600 HU) and a soft tissue algorithm (W400; L90 HU). The voxel size ranged between 0.4883 × 0.4883 × 1.5 mm to 0.5859 × 0.5859 × 1.5 mm, depending on the field of view.

### Measurement Protocol

**Figure 1:**
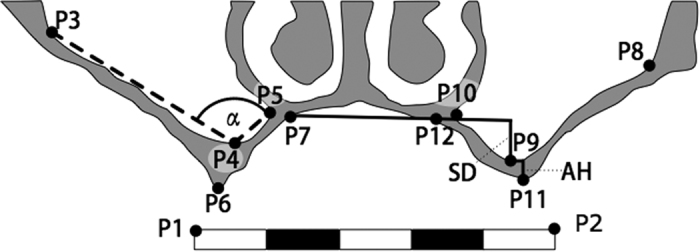
Measurement Protocol: Placement of Landmarks P1–P12; Calculation of sinus depth (SD) and alveolar height (AH) are illustrated in the left sinus, calculation of the sinus opening angle (α) is shown in the right sinus. The palatal plane is marked as horizontal black line between P7 and P12.

All of the measurements were performed in the coronal bone window at height of the zygomaticoalveolar crest. After identification of this structure the corresponding layer was extracted from the database of the Department of Radiology and saved as. jpeg (Joint Photographic Experts Group) file on an external hard drive. All of the measurements were performed using a java – based program specially designed for this purpose by one of the authors (RS). The measurement procedure always followed the same routine: In a first step twelve reference points were placed manually (see Fig. 1). The first two points (P1, P2) were placed as calibration points on both ends of a digital ruler in each radiograph. Next, three reference points were placed inside the right sinus (P3–P5); the first point (P3) was placed at the highest point of the lateral sinus wall, the second point (P4) was placed at the lowest possible point of the sinus floor, and the third point (P5) at the highest point of the medial sinus wall. Finally, another point (P6) was placed at the alveolar tip and at the height of the hard palate (P7). The same sequence was followed at the patient’s left sinus (P8–P12). All of the radiographs were measured twice by two independent observers with an interval of six weeks between the measurements (total: 12 points x 400 patients x 2 measurements x 2 observers = 19200 reference points). The median was computed out of these outcome four-tuples and deviations >2 mm were identified as measurement errors and adjusted until consensus was reached. The double – checked points were then used for morphometric analysis.

### Morphometric Analysis and Statistics

The alveolar height (AH) was calculated as difference between the alveolar tip (see Fig. 1: right P6, left P11) and the deepest point of the sinus floor (right P4, left P9). Sinus depth (SD) was computed from the deepest sinus point (right P4, left P9) and its projection on the hard palate (plane through points P7 and P12). The sinus-opening angle (see Fig. 1: α) was calculated from the deepest sinus point (right P4, left P9) and the highest possible points for the palatal (right P5, left P10) and the buccal (right P3, left P8) sinus wall.

Morphometric and statistical analysis were conducted with the open source statistical program “R” (version 2.15.1, http://cran.r-project.org). A sinus classification was built on the first and the third quartile of the sinus depth (projection of the sinus into the alveolar recessus as defined above) stratified by the group (edentulous vs. dentate). All parameters were reported as mean ± standard deviation. Sinus depths were compared regarding dentition group and gender using analysis of variance (ANOVA: Model 1: sinus depth ~ dentition group; Model 2: sinus depth ~ gender). Alveolar height was analysed regarding the three sinus classes (first quartile, second + third quartile, and fourth quartile), the dentition group, and gender by means of ANOVA (Model 1: alveolar height ~ sinus class; Model 2: alveolar height ~ dentition group; and Model 3: alveolar height ~ gender). Similarly the sinus opening angle of sinus classes (Model 1: sinus opening angle ~ sinus class), gender (Model 2: sinus opening angle ~ gender), and dentition group (Model 3: sinus opening angle ~ dentition) were compared by means of ANOVA. Multivariate ANOVA (MANOVA) was carried out to compensate for multiple testing (Model 1: sinus depth ~ dentition group + gender, Model 2: alveolar height ~ sinus class + dentition group + gender, Model 3: sinus opening angle ~ sinus class + dentition group + gender).

## Results

The authors reviewed 4837 CT scans that were performed between October 2007 and November 2013 in order to reach the number of 200 edentulous and 200 dentulous CT scans meeting the inclusion criteria. A total of 4437 CT scans were excluded due to pathologic lesions in the maxillary sinus region, or since they did not display the entire maxillary sinus or had missing bone window reconstructions. In the course of the landmarking process another six CT scans (1.5%: 4 male with teeth, 1 edentulous male, 1 edentulous female) were excluded due to indistinguishable sinus bone contours, resulting in 788 sinuses for final morphometric evaluation.

### Sinus classification regarding sinus depth

**Figure 2:**
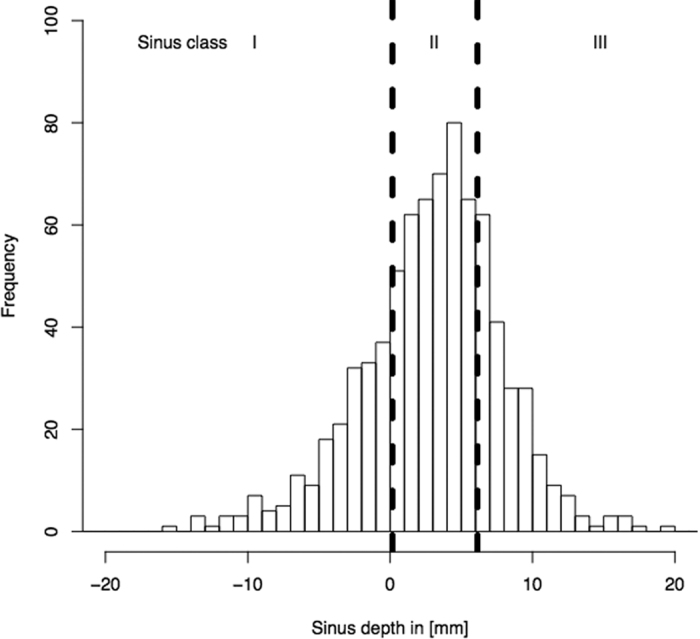
Histogram of the sinus depth. Vertical lines resemble the first and the third quartile used as class limits. The first quartile was 0 mm and the third quartile 6 mm. The first class has a sinus floor above the hard palate, the second class’ floor is 0 to 6 mm below and the third class is more than 6 mm below the hard palate. The first class resembles 25%, the second class 50% and the third class 25% of the patients.

**Figure 3:**
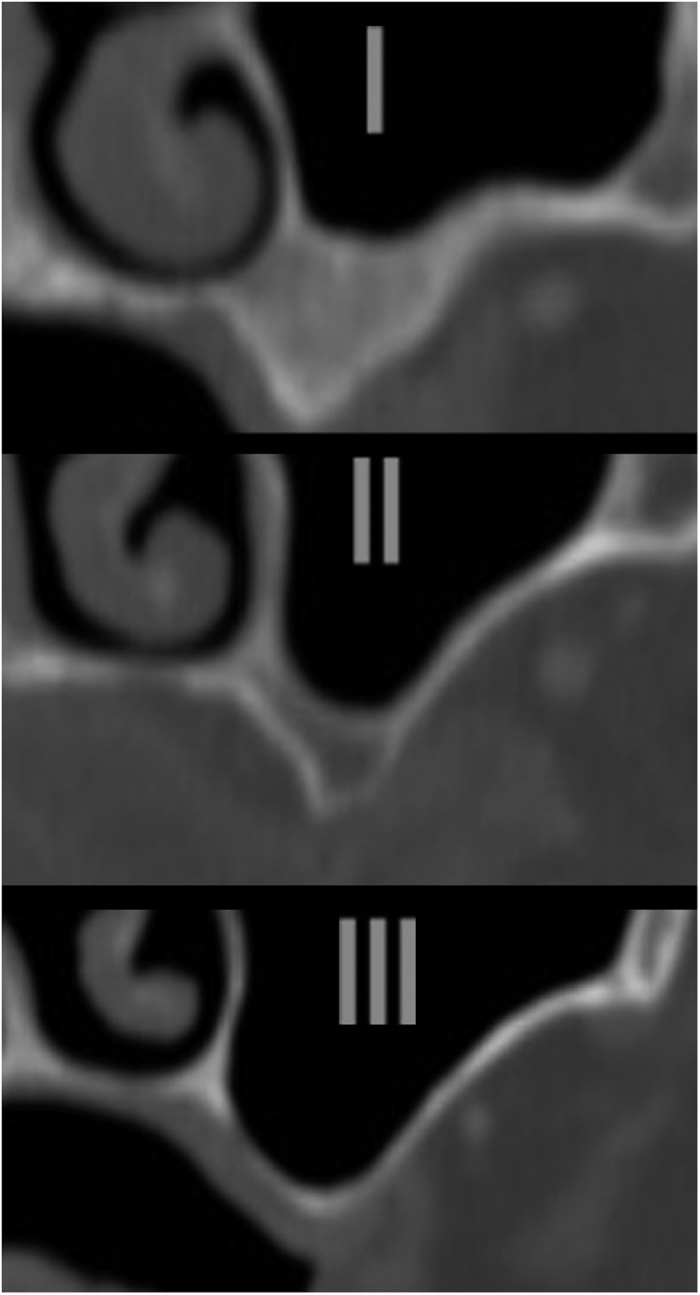
Three reference sinuses corresponding to the three newly defined sinus classes are displayed.

A reasonable classification was built upon the rounded quartiles of sinus depth (see Fig. 2). The first quartile was 0.3 mm in dentulous and 0.0 in edentulous patients (rounded to 0) and the third quartile 5.9 in dentulous and 6.2 mm in edentulous patients (rounded to 6 mm). Equally, the first quartile was 0.1 mm in male and 0.2 mm in female patients, the third quartile was 6.0 in male and 6.2 in female patients. Sinuses are thus to be divided according to their extension into the alveolar process measured from the hard palate (see Fig. 3): either above (I), 0–6 mm below (II), or more than 6 mm below (III). Therefore, in 25% the sinus floor was found to be located above the palatal plate (class I), in 50% the sinus floor was 0–6 mm below the palatal plate (class II) and in 25% the sinus floor was found to be ≥6 mm below the palatal plate (class III). Sinus depth did not vary significantly between gender (female: 3.1 ± 5.44 mm, male: 2.96 ± 5.41 mm, ANOVA: F = 0.0178, DF = 1, p = 0.8939; MANOVA: F = 0.0178, p = 0.8940) or dentition groups (dentate: 3.1 ± 5.6 mm, edentulous: 2.9 ± 5.3 mm, ANOVA: F = 0.0011, DF = 1, p = 0.9734; MANOVA: F = 0.0012, p = 0.9723). Mean age through the three newly defined sinus classes did not vary significantly (class I: 57.3 ± 17.5, n = 197; class II: 57.0 ± 16.0, n = 394; class III: 58.2 ± 16.7, n = 197 ANOVA: F = 0.3013, p = 0.583).

### Alveolar Height

**Figure 4:**
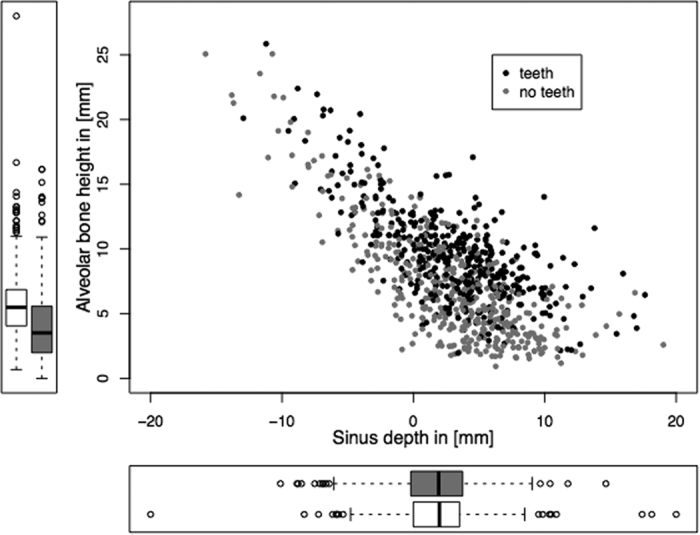
Scatterplot of the sinus depth versus the alveolar height stratified by the dentition group. Marginal boxplots for the groups restrict the information to either sinus depth or alveolar height. Sinus depth gradually increases with decreasing alveolar bone height. Dentition groups did significantly influence alveolar bone height but not sinus depth.

The alveolar height was 13.0 ± 4.9 mm (range: 2.2 to 42.8) in class I, 7.7 ± 2.9 mm (range: 1.7 to 17.1) in class II, and 5.44 ± 2.52 mm (range: 0.9 to 14.0) in class III. The sinus classes showed a significantly different alveolar height (ANOVA: F = 258.34, DF = 2, p < 2.2 × 10^−16^; MANOVA: F = 301.0573, p < 2 × 10^−16^). Alveolar height differed significantly between the dentition groups (see Fig. 4, dentate: 9.7 ± 4.1 mm, edentulous: 7.1 ± 4.3 mm; ANOVA: F = 72.051, DF = 1, p < 2 × 10^−16^, MANOVA: F = 131.3715, p < 2 × 10^−16^) but not between gender (female: 8.3 ± 4.4 mm, male: 8.5 ± 4.4 mm; ANOVA: F = 0.4969, DF = 1, p = 0.4811; MANOVA: F = 0.4187, p = 0.5178).

### Sinus opening angle

**Figure 5:**
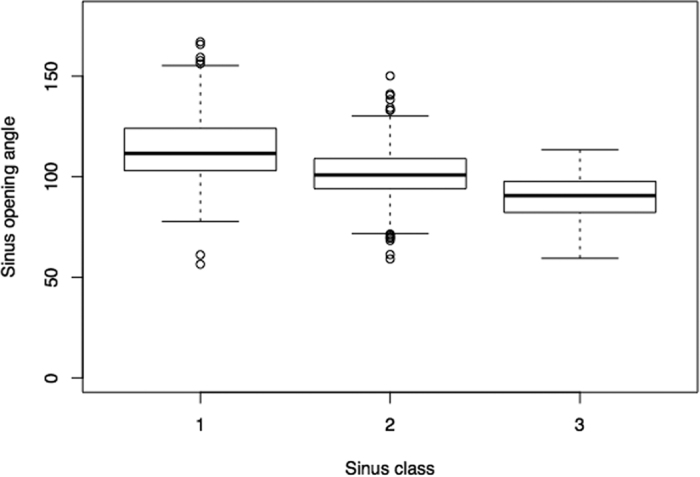
Boxplot of sinus opening angle stratified by sinus class. The mean opening angle was significantly different between the sinus classes: 113.9 ± 17.6 in class I, 101.2 ± 13.3 in class II and 90.1 ± 10.7 in class III.

The sinus opening angle was compared between the newly defined subclasses; the mean opening angle in class I was 113.87 ± 17.56 (ranging from 56.5 to 167.11), 101.19 ± 13.32 (range: 59.1 to 150.15) in class II and 90.13 ± 10.73 (range: 59.4 to 113.49) in class III (see Fig. 5). The sinus classes showed significantly different sinus opening angles (ANOVA: F = 144.32, DF = 2, p < 2.2 × 10^−16^; MANOVA: F = 145.0775, p < 2.2 × 10^−16^). Neither the dentition groups (dentate: 100.3 ± 15.7, edentulous: 102.4 ± 16.6; ANOVA: F = 3.3197, p = 0.06883; MANOVA: F = 5.2521, p = 0.02218) nor gender (female: 101.6 ± 15.2, male: 101.1 ± 17.2; p = 0.650; ANOVA: F = 0.2063, p = 0.6498; MANOVA: F = 0.8884, p = 0.34620) did significantly differ regarding the sinus opening angle.

## Discussion

Existing alveolar ridge and sinus classifications focused on various anatomical and radiological landmarks but did not consider the extension of the maxillary sinus into the alveolar process, which is of crucial knowledge in sinus augmentation procedures^1^,^2^,^3^,^5^,^7^,^8^,^9^. Hence, the aim of this study was to investigate the extension of the maxillary sinus into the alveolar process and to provide a simple and easily clinically applicable classification.

Sinuses were divided into three newly defined anatomical classes according to the quartiles of sinus depth: class I (above the hard palate), class II (0–6 mm below the hard palate) and class III (>6 mm below the hard palate). Consistent class limits were found for edentulous and dentate groups. The sinus classification was not depending on gender or dentition group. The alveolar height was significantly influenced by the sinus class (i.e. the extension of the sinus into alveolar process, commonly known as the alveolar recessus) and the dentition group but not by gender.

Sinus depth and remnant alveolar height are important anatomical parameters that have to be scrutinized rigorously by the clinician in the course of preprosthetic treatment planning. Depending upon the sinus class, different treatment regimes may be at disposal: in sinuses belonging to class I, with little sinus depth and only slightly reduced RH, implants may usually be placed without prior sinus augmentation. This clinically easy class might be expected in a quarter of the patients without the need for sinus augmentation. If the alveolar ridge was totally lost, and - despite the high sinus floor - sinus augmentation is needed prior to implant placement, sinus augmentation has to be performed via the conventional lateral approach and not the palatal approach. In these cases, the position of the sinus above the level of the hard palate has to be taken into consideration for the adequate position of the buccal window. In sinuses belonging to class II and III, with greater sinus depth and moderately to severely reduced RH, sinus augmentation is indicated prior to implant placement unless ultra short implants are being used. Especially class III sinuses however are predestined for palatal sinus lifting^11^,^12^,^13^. Thus, this sinus classification offers a clear treatment guide regarding the anatomical aspects of the maxillary sinus. Class I mostly doesn’t require sinus lifting, class III is open to all approaches (lateral, transcrestal, palatal) and class II is better to be done from a lateral window or using special transcrestal lifting equipment for hydraulic detachment of the Schneiderian membrane (such as a balloon or the Jeder system) if the remnant height of the alveolar ridge requires long transalveolar working channels^14^,^15^,^16^.

The dentition group did only show effects in the multivariate analysis, which might implicate an interaction of sinus class and dentition group. As mentioned above, no significant differences in sinus depth between gender or dentition groups were found, implicating that sinus depth might be considered a constitutional trait. Sinus depth thus represents a stable and appropriate variable to build this classification, applicable in patients independent of gender and dentition. This sets this novel classification apart from other pre-existing classifications, as potential differences between genders have not been considered and measurements were only performed in the edentulous jaw^1^,^2^,^3^,^5^,^6^,^9^.

Alveolar height was significantly influenced by the sinus class: the average residual ridge height (RH) gradually decreased with increasing sinus depth from class I to class III. This can be attributed to the greater extension of the maxillary sinus into the alveolar process, inevitably resulting in a reduction of the RH.

A significant influence of dentition on the alveolar height was revealed by statistical analysis, while the effect of gender did not contribute to significant differences. This finding is in agreement with the literature: prolonged edentulism leads to ongoing residual ridge resorption (RRR) due to reduced masticatory forces^17^,^18^,^19^. Findings regarding the effect of gender on RRR and RH are scarce and discussed controversial in the literature. While some studies found significant differences in bone loss between genders others were not able to confirm these findings^20^,^21^,^22^.

One of the most common complications during sinus augmentation procedures is perforation of the Schneiderian membrane; incidence rates for the lateral approach vary between 8.6% and 41% in the literature^23^,^24^,^25^. While recent studies found that the overall implant survival rate is not affected by membrane perforations, patients still face the increased risk of postoperative inflammation and graft failure as well as delay of prosthetic rehabilitation^23^,^25^. Certain anatomical features, such as presence of sinus septa, narrow sinuses and sharp angles of the sinus walls have been found to increase the risk of membrane perforation^26^,^27^,^28^.

As several other classifications are based on sinus width we chose to calculate the sinus-opening angle in the present study in order to allow for direct comparison^2^,^5^. Sinus opening angles in this study were found to be significantly different between sinus classes but not between gender and dentition. The average sinus opening angle was the largest in class I and gradually decreased from class I to class III. Our findings are in contrast to the findings of Cho *et al*. and Velloso *et al*., who found considerably smaller angles between the medial and lateral sinus wall in study groups of 49 and 26 sinuses, respectively. Although Cho *et al*. reported a membrane perforation rate of 37.5% in sinuses with opening angles ≤30°, they found a rate of 0% in sinuses with an opening angles ≥61°. This indicates a very low risk of membrane perforations throughout all of the three newly defined sinus classes in this study.

Besides the retrospective nature of this study several limitations have to be mentioned; information regarding systemic conditions, medication intake, duration of edentulism as well as use of dentures was not available. Finally, only two-dimensional changes in sinus depth were examined.

## Conclusion

A novel classification, dividing the sinuses into three newly defined anatomical classes according to sinus depth was built: class I (above the hard palate), class II (0–6 mm below the hard palate) and class III (>6 mm below the hard palate). This simple and easily clinically applicable classification adds a novel aspect to existing classifications and represents the anatomy independent of gender and dentition of the individual. It aims to guide clinicians during preprosthetic treatment planning and to facilitate communication between them. Future studies in the third dimension are planned to test the validity of the proposed classification.

## Additional Information

**How to cite this article**: Wagner, F. *et al*. Morphometric analysis of sinus depth in the posterior maxilla and proposal of a novel classification. *Sci. Rep.* **7**, 45397; doi: 10.1038/srep45397 (2017).

**Publisher's note:** Springer Nature remains neutral with regard to jurisdictional claims in published maps and institutional affiliations.
